# Shade affects magnitude and tactics of juvenile Chinook salmon antipredator behavior in the migration corridor

**DOI:** 10.1007/s00442-021-05008-4

**Published:** 2021-08-05

**Authors:** Megan C. Sabal, Michelle L. Workman, Joseph E. Merz, Eric P. Palkovacs

**Affiliations:** 1 Department of Ecology and Evolutionary Biology, University of California Santa Cruz, 130 McAllister Way, Santa Cruz, CA 95060 USA; 2 East Bay Municipal Utility District, 1 Winemaster Way, Lodi, CA 95240 USA; 3 Cramer Fish Sciences, 3300 Industrial Blvd #100, West Sacramento, CA 95691 USA

**Keywords:** Movement, Predation risk, Light, Structure, Refuge

## Abstract

Environmental conditions strongly affect antipredator behaviors; however, it is less known how migrating prey adjust antipredator behavior in migration corridors, in part, because active migrants are difficult to observe and study. Migrants are vulnerable and encounter many predators in the corridor, and their propensity to travel towards their destination ties antipredator behavior with movement. We evaluated how environmental risk cues in the migration corridor including in-water habitat structure (present, absent) and overhead shade (sun, shade), and salmon origin (hatchery, wild) affected how juvenile Chinook salmon (*Oncorhynchus tshawytscha*) reacted to a live predator. We measured how salmon react to predation risk as the difference in time to swim downstream through a 9.1-m long field enclosure with or without a live predatory largemouth bass (*Micropterus salmoides*). Shade significantly modified the reaction to the predator, and it did so in two ways. First, the magnitude of antipredator behavior was larger in shade compared to direct sun, which suggests salmon perceived shade to be a riskier environment than sun. Second, the escape tactic also varied; salmon slowed down to be cautious in shade and sped up in sun. Structure did not significantly affect behavior and hatchery and wild salmon behaved similarly. Our study suggests that environmental risk cues can shape the magnitude and tactics of how migrants react to predation risk and illustrates how these responses relate to movement with potential to scale up and affect migration patterns.

## Introduction

Prey use environmental cues to assess predation risk and adjust their antipredator behavior (Lima and Dill 1990). For example, animals often perceive sheltered habitats to be less risky than exposed habitats, and consequently, in sheltered habitats prey allow predators to approach more closely before they flee (de Boer et al. 2004). Habitat-dependent antipredator behavior has been heavily studied in resident prey and can scale up to affect populations and communities (Preisser et al. 2005; Wirsing et al. 2021). However, these antipredator decisions are much less studied in migrating animals, despite that migrants often experience high predation risk in migration corridors where they are concentrated and conspicuous (Furey et al. 2018). Furthermore, antipredator behavior in the migration corridor affects fine-scale movement creating a potential pathway to affecting migration duration or arrival time (Hope et al. 2014; Sabal et al. 2020). Migratory prey are economically and ecologically valuable and, thus, it is important to examine their context-dependent antipredator behavior in the risky migration corridor.

Habitat structure is a powerful environmental driver of prey antipredator behavior. Structure commonly decreases predation rates because it disrupts visual hunting by predators and provides prey refuge (Crowder and Cooper 1982; Allouche and Gaudin 2001; Bonnot et al. 2016). Consequently, animals engage in less antipredator behavior when closer to refuge (e.g. wait longer to flee from an approaching predator, be less vigilant) (Frid 1997; Stellatelli et al. 2015). Structure can also cause prey to shift escape tactics. For example, bluegill sunfish (*Lepomis macrochirus*) evaded largemouth bass (*Micropterus salmoides*) by hiding when structure was present and by schooling when structure was absent (Savino and Stein 1982). Therefore, structure may influence the magnitude and escape tactics of migrating prey. However, in migration corridors local habitat structure is less familiar to transient migrants compared with local habitats of resident prey, which could alter migrant behavioral responses to structure in the corridor (Forrester et al. 2015; Moore 2018).

Shade during daylight influences antipredator decisions commonly across taxa and is relevant to antipredator decisions on a similar scale as habitat structure. For many organisms, shade indicates low risk because it provides cover, is often correlated with structure, and reduces glare, which improves detection of predators (McMahon and Hartman 1989; McCartt et al. 1997; Mandelik et al. 2003; Carr and Lima 2014). Therefore, we might expect prey to exhibit lower intensities of antipredator behavior in shade. For example, house finches (*Carpodacus mexicanus*) perceived shade to be less risky than sunlight and reduced their antipredator response to predation risk in shade (e.g. reaction time, scanning behavior) (Fernández-Juricic and Tran 2007). However, shade may also indicate high risk. Overhead shade that is not associated with structure, including anthropogenic structures (e.g. docks, bridges), may cause prey avoidance in fish (Able et al. 2013; Nebel et al. 2019). Additionally, low light can decrease prey vision and reduce information about the predator and environment, increasing perceived risk (Cerri 1983; Leahy et al. 2011). Migrants encounter many predators across an environmentally-variable migration corridor with shade from natural and anthropogenic sources with the potential for shade to indicate low or high risk.

Juvenile Pacific salmon (*Oncorhynchus* spp*.*) encounter numerous predators as they migrate from freshwater to marine environments (Rieman et al. 1991; Evans et al. 2012; Thomas et al. 2017). When rearing upstream, salmon use structure and shade from riparian vegetation, which often covers much of the stream channel, to hide from predators (McMahon and Hartman 1989; Reinhardt and Healey 1997; Korstrom and Birtwell 2006; Penaluna et al. 2015). However, when salmon migrate through the larger rivers of the migration corridor, the role of habitat mediating predation risk is unclear. Habitat structure, including woody debris and submerged aquatic vegetation, has been presumed to both decrease predation risk by providing prey refuge and increase predation risk by providing predator habitat (Zajanc et al. 2013; Henderson et al. 2019). Migrating juvenile salmon also can have a variable reaction to shade—they may avoid passing through shade cast by anthropogenic structures, but also preferentially hold in shaded reaches with complex habitat (Kemp et al. 2005; Zajanc et al. 2013; Ono and Simenstad 2014; Hellmair et al. 2018). Habitat structure and shade are more constrained to the shoreline in the migration corridor.

An additional element likely influencing juvenile salmon antipredator behavior is the role of fish hatcheries. Hatchery salmon often differ in genetics, morphology, physiology, and behavior compared to salmon born in the river (“wild salmon”) (Weber and Fausch 2003; Jonsson and Jonsson 2006). Hatchery salmon lack prior predator exposure, which may cause them to react less to predation risk than wild salmon (Alvarez et al. 2003; Roberts et al. 2011). Overall, migrating juvenile salmon encounter many predators and habitats potentially associated with different risk levels, while hatchery influence may further affect salmon antipredator behavior.

To examine antipredator decisions in the migration corridor, we need to measure a behavioral reaction to predation risk and observe how that reaction changes under different contexts. When prey react more strongly to a predator, for example in exposed compared to sheltered habitats, we can assume the prey perceived higher predation risk (Ydenberg and Dill 1986; Camp et al. 2012). Since migrating prey have a propensity to travel in a set direction, we can assess their reaction to predation risk by measuring how much they adjust their travel speed (or time to destination) to engage in antipredator behavior (Sabal et al. 2020). Cryptic and cautious antipredator behavior in migrants slows travel speed—for example, increasing vigilance, hiding in cover, and punctuated movements (Chung et al. 2009; Hope et al. 2014; Melnychuk and Welch 2018). Alternatively, migrants may speed up to reduce encounter time with comparatively stationary predators (Peterson 1976; Proffitt et al. 2009). Therefore, how much prey adjust their travel speed indicates the magnitude of perceived predation risk, while the direction that prey change their travel speed relates to their escape tactic.

We performed a behavioral assay with juvenile Chinook salmon (*O. tshawytscha*) to measure the difference in downstream travel speed with and without a predatory largemouth bass (*Micropterus salmoides*) present (i.e., their reaction to a predator). We observed two aspects of the antipredator response—the magnitude (amount of perceived predation risk) and direction (escape tactic) of the change in travel speed. We evaluated context-dependence of salmon antipredator responses by varying structure (present, absent), shade (sun, shade), and salmon origin (hatchery, wild). Specifically, we asked (1) do migrating salmon change their travel speed in response to the presence of a predator, and (2) how do structure, shade, and salmon origin modify the magnitude and direction of their change in travel speed?

We predicted that salmon would change their travel speed to engage in antipredator behavior in response to predator presence. For the magnitude of antipredator response, we predicted that structure and shade would modify perceived predation risk. However, we did not make an a priori prediction as to whether the presence of structure or shade would increase or decrease the reaction to the predator because of conflicting prior evidence and the knowledge that animals in the migration corridor may respond to environmental risk cues differently than when not migrating (Zajanc et al. 2013; Ono and Simenstad 2014; Henderson et al. 2019). We predicted that wild salmon would react more strongly to a predator compared to hatchery salmon because of increased prior predator experience (Roberts et al. 2011; Solberg et al. 2020). We did not make an a priori prediction as to the escape tactic because salmon may slow down to evaluate risk or speed up to reduce encounter time with relatively stationary predators (Petersen and Deangelis 2000; Vehanen 2003; Sabal et al. 2020).

## Materials and methods

We performed 144 behavioral assays on juvenile fall-run Chinook salmon between 02-May-2018 and 31-May-2018. We measured the magnitude of antipredator behavior as the difference between the mean travel speed without the predator and the mean travel speed with the predator present. The difference in travel speed can be caused by various antipredator behaviors, such as fleeing, predator inspection, reducing activity, and temporarily hiding (Sabal et al. 2020). Trials occurred on the lower Mokelumne River, California—a part of the migration corridor.

### Study site

The experiment’s location on the lower Mokelumne River (38.202957° N − 121.516989° W) sits approximately 41 river-km downstream from salmon spawning locations and 130 km upstream of the Pacific Ocean. When migrating juvenile salmon reach the study area, they encounter relatively low water velocities that change direction due to tidal influence (range − 0.3 to 0.4 m/s). We only performed trials during outgoing tides when salmon are more likely to travel downstream (Hering et al. 2010). Juvenile Chinook salmon have a propensity to migrate nocturnally; however, diel movements also occur and increase in frequency in the Sacramento-San Joaquin Delta compared to upstream reaches (Chapman et al. 2012). River turbidity at our study site was relatively low (mean + sd: 3.48 ± 1.13 NTUs) and likely would not strongly affect reaction distances between predator and prey (Miner and Stein 1996). The river is bordered by leveed banks with riprap and occasional trees, which cast shade onto the river margins on clear days. Woody debris and submerged aquatic vegetation are occasionally present along this stretch of the lower Mokelumne River.

### Juvenile salmon and largemouth bass

We used hatchery and wild juvenile Chinook salmon to examine how salmon origin affects antipredator decisions. Hatchery salmon were obtained from the Mokelumne River Fish Hatchery where hatchery and wild salmon are managed as a single gene pool (Williamson and May 2005). Therefore, any behavioral differences in hatchery salmon in our experiment are presumably from altered rearing conditions. Wild salmon were obtained from a rotary screw trap (2.4 m in diameter; E.G. Solutions, Inc.) operated by East Bay Municipal Utility District. The screw trap was operated 35 river km upstream of the study site and only captures actively migrating salmon. Both wild and hatchery salmon were obtained weekly and held in coolers with aeration on site until they were run in trials (days since capture, range: 0–4 days). Largemouth bass (*n* = 19) were obtained from nearby locations through boat electrofishing and hook-and-line sampling and were held in 1135 L-tanks on site for the entire month of experiments. We weighed, measured fork length (FL), and calculated Fulton’s condition factor (*K*) of juvenile salmon. Largemouth bass FL ranged from 198 to 326 mm and were all capable of consuming juvenile salmon (66–109 mm FL) (Hambright 1991; Michel et al. 2018). External anchor tags (Floy Tag, Inc.) were used to track individual bass while internal Passive Integrated Transponder (PIT) HDX12 PIT tags were used to identify individual juvenile salmon. Prior to tagging, salmon were anesthetized in a dose of MS-222 (0.2 g/L) buffered with NaHCO_3_ (2 g/L) (Topic Popovic et al. 2012). All salmon were well below the 6.7% tag burden limit which may affect survival (Brown et al. 2010).

### Enclosures

**Fig. 1:**
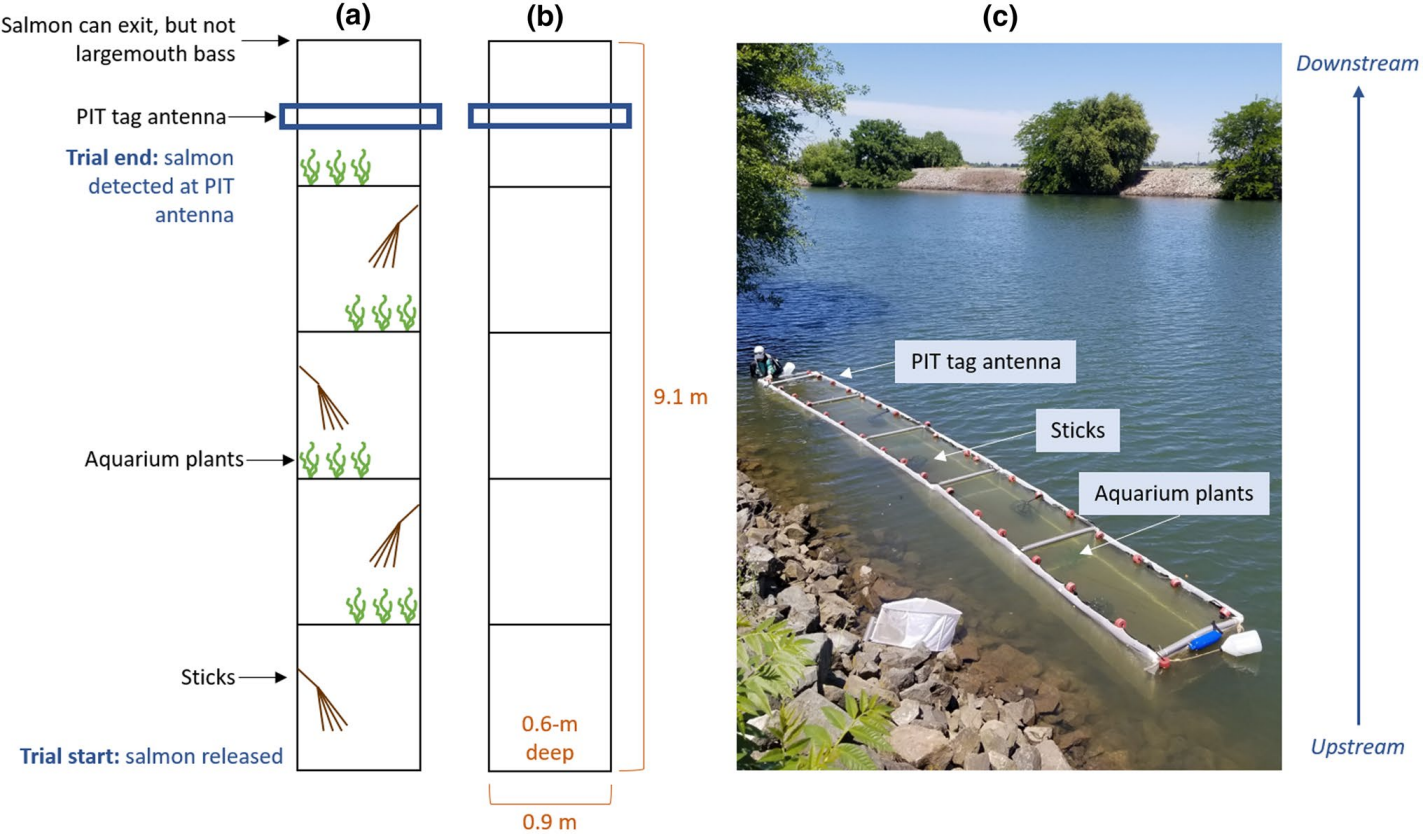
Diagram of enclosure set up, **a** enclosure with structure, **b** enclosure without structure, **c** photograph of enclosure with structure in sun

To measure salmon downstream travel speed, we built two rectangular floating field enclosures that were 9.1-m long, 0.9-m wide, and 0.6-m deep (Fig. 1). The frame was built of Polyvinyl chloride (PVC) pipe and enclosed with white 0.3 cm^2^ mesh netting on all sides except for the top, which was covered with 2.5 cm^2^ bird netting. The downstream end also did not contain mesh and instead was covered with 3 cm^2^ wire grid to allow salmon to exit into the river but prevent largemouth bass from escaping. The top PVC pipe was surrounded by pipe insulation, allowing the enclosures to float on the water surface with the long sides parallel to the shoreline. The enclosures were held in place by anchored buoys at two specific locations near the shoreline. We placed a PIT tag antenna around the outside of the enclosure frame one meter up from the downstream exit. Placement in front of the exit was to detect downstream movement before salmon may have started to exhibit caution due to approaching a novel environment (i.e. latency to exit). The enclosure design precluded some juvenile salmon natural antipredator behaviors, such as schooling and movements perpendicular to flow, but did allow predator inspection, vigilance, and cautious behavior, which are common fish antipredator behaviors over small spatial and temporal scales (Kelley and Magurran 2003).

### Habitat structure

To test if structure modified salmon risk perception, we kept one enclosure empty and the other included habitat structure (Fig. 1). To imitate submerged aquatic vegetation, three plastic aquarium plants with three stems per plant (height: 0.27 m) were attached to the enclosure bottom at each of four equidistant points from the upstream to downstream length of the enclosure. To represent woody debris and overhanging vegetation, four 38-cm tall, 22-cm diameter cylinders of wire grid (2.5 cm^2^) encased 5–10 sticks and were attached to the upper PVC frame and entered the enclosure from above. The four habitat cylinders were evenly distributed throughout the enclosure and alternated sides. We rotated the enclosures with and without habitat structure between locations each week. Submerged aquatic plants and overhead sticks are common forms of shoreline habitat structure in this study reach (M. Sabal pers. obs.).

### Shade

**Fig. 2:**
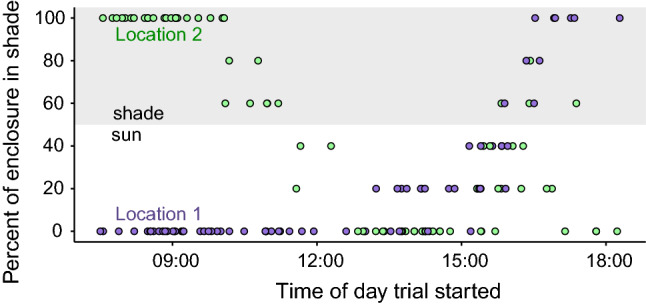
Shade and sun regimes over time of day at each enclosure location: location 1 (purple) and location 2: (green). Shade trials occurred when the enclosure was 60, 80, or 100% in shade, while sun trials were when the enclosure was 0, 20, or 40% in shade (colour figure online)

To test if shade modified salmon antipredator decisions, we recorded if behavioral trials occurred in shade or direct sunlight. All trials occurred during daylight between 7:30 (90 min after sunrise) and 19:00 (60 min before sunset) on non-overcast days. Each enclosure location experienced different shade regimes from nearby trees on the levee casting shade onto one enclosure in the afternoon (after 16:00) and onto the other enclosure in the morning (before 11:00) (Fig. 2). Different shade regimes between the two locations helped disentangle the shade effect from time of day. The enclosures were visually broken up into five segments by structural PVC (Fig. 1). We noted how many sub-sections were more than 50% shaded and recorded these as the percent of enclosure shaded (possible values were 0, 20, 40, 60, 80, and 100%). To maintain sufficient sample sizes within shade categories for our analyses, we grouped the six percent shade categories into two categories: shade and sun. Trials were categorized as shaded when three or more sub-sections were shaded (60, 80, or 100% of the total enclosure), and trials were categorized as in sun when two or less sub-sections were shaded (0, 20, or 40% of the total enclosure).

### Behavioral assay protocol

We ran behavioral assays, timing how long it took juvenile Chinook salmon to swim downstream through an enclosure with and without a largemouth bass. The difference in travel time between predator treatments represents salmon’s behavioral reaction to predation risk. For predator trials, one largemouth bass was transported to the field enclosure and acclimated to the enclosure and river water for 30 min. For all trials, one juvenile salmon was transported to an acclimation container attached inside at the upstream end of the enclosure and acclimated to the river water for 10 min. To begin a trial, the container was opened and rotated to release the salmon into the enclosure, and this start time was recorded. The trial ended when the salmon reached the PIT tag antenna at the end of the enclosure or after one hour if never detected. Largemouth bass commonly swam throughout the enclosure, but we did not measure specific bass behavior. At the trial’s end, we pulled a crowder through the enclosure to ensure the salmon exited and to recapture the largemouth bass. Only one salmon escaped the enclosure during a trial—all other salmon were accounted for at the end of the trial. An individual largemouth bass remained in the enclosure for two subsequent trials and then was removed. We rotated between two trials with a predator present and two trials with no predator, and we staggered which type of trial started in each enclosure. Each largemouth bass was only used once per week in two subsequent trials. We also staggered which enclosure started with hatchery or wild salmon and rotated every trial between each prey origin. All handling and procedures were approved under Institutional Animal Care and Use Committee (IACUC) protocol PALKE1701.

### Analyses

To examine context-dependent salmon antipredator behavior in the migration corridor, we observed if travel speed was influenced by the interaction between predator presence and habitat structure, shade, and salmon origin. We used a mixed-effects Cox regression because it uses censored data over time—both measures of an event (salmon reaching the PIT antenna) happening or not (0,1) and the time at which that event occurred (R package: ‘coxme’; Therneau 2015). Therefore, the Cox model response represents travel speed, as it measures the probability of reaching the antenna (traveling a set distance) over time and accounts for salmon that failed to reach the PIT antenna in the allotted 60 min. The preferred method is to include subjects with censored times in analyses because they represent important biological observations—the individuals with the most extreme behaviors (Fox 2015). Cox regressions appropriately consider those observations as censored and not as observations occurring at the cut-off time.

We included in the Cox model as main effects: predator presence (present, absent), structure (present, absent), shade (sun, shade), and salmon origin (hatchery, wild). We included all two-way interactions to assess how structure, shade, and salmon origin interact with predator presence and to account for other potential behavioral interactions among covariates. We included enclosure location as a random effect. We subsequently performed a Type III ANOVA on the Cox model to assess covariate significance. We used linear contrasts on the Cox model (R package: ‘lsmeans’; Lenth and Love 2017) and effect sizes on log-transformed time to finish (Hedge’s *g*; R package ‘effsize’; Torchiano 2017) to determine post-hoc differences in reaction to predation risk among structure, shade, and salmon origin. Although unrelated to our specific hypotheses, we also tested if salmon traits (FL, weight, K condition factor) or experimental attributes (time of day, date, days since salmon capture) affected the reaction of salmon to the predator. We used separate mixed-effects Cox models for each variable and related salmon travel speed to the interaction of predator presence and the variable of interest with location as a random effect, and subsequent Type III ANOVA tests.

## Results

We performed 144 behavioral assays. The treatments we manipulated (predator presence, structure, and salmon origin) were approximately evenly distributed across total trials (after excluding overcast days). However, shade varied naturally, and treatments were uneven across shade and sun (Table 2). Water velocity inside the enclosure (mean + sd: 0.02 ± 0.01 m/s) was relatively low and consistent over the course of the experiments regardless of tidal stage. Hatchery and wild juvenile salmon varied over a similar size range (hatchery: 74–96 mm, wild: 72–109 mm FL). A summary of hatchery salmon physical attributes were (mean + sd: FL: 83.7 ± 4.6 mm, weight: 6.9 ± 1.2 g, *K*: 1.16 ± 0.06) and wild salmon attributes (FL: 80.8 ± 8.0 mm, weight: 7.6 ± 2.2 g, *K*: 1.02 ± 0.05). Neither salmon traits or experimental variables significantly influenced the reaction of salmon to the predator (Type III ANOVAs, *n* = 144: salmon FL [*χ*^2^ = 0.90, *p* = 0.34], salmon weight [*χ*^2^ = 0.88, *p* = 0.35], salmon *K* [*χ*^2^ = 0.01, *p* = 0.90], time of day [*χ*^2^ = 0.39, *p* = 0.53], date [*χ*^2^ = 0.53, *p* = 0.47], days since salmon capture [*χ*^2^ = 0.92, *p* = 0.34]).

**Fig. 3:**
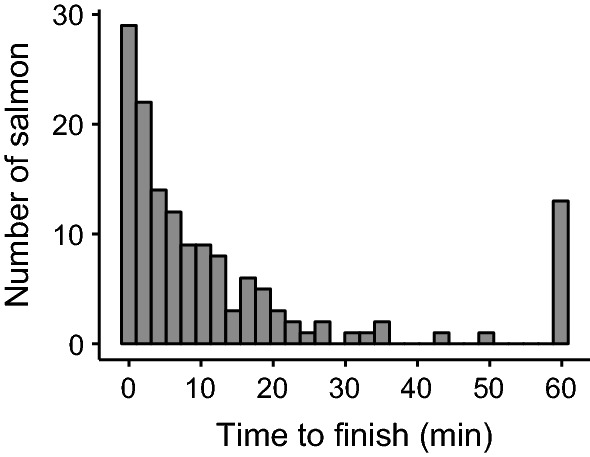
Histogram of time to reach the end of the enclosure (min) across all salmon (*Oncorhynchus tshawytscha*) trials. Salmon binned at 60 min did not finish in the allotted 60 min. Salmon with censored times were not considered as 60 min in analyses, but instead were appropriately included in Cox regressions as censored values

**Fig. 4:**
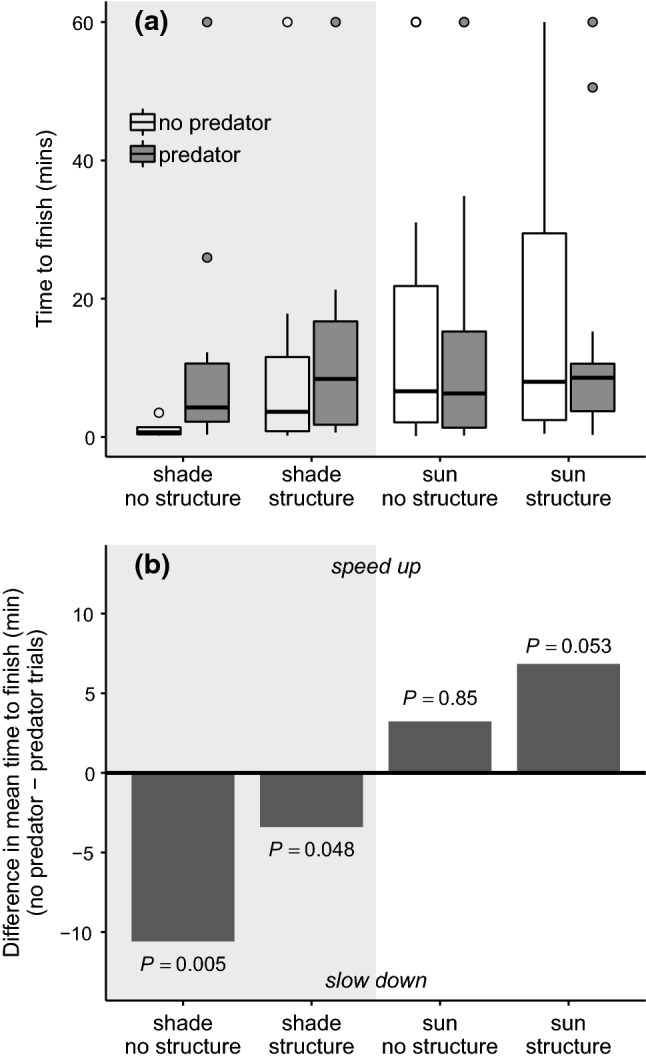
**a** Boxplot of salmon’s time to finish (min) for each habitat treatment combination of shade and structure, by predator treatment (gray = present, white = absent). Whiskers represent 95% confidence intervals. **b** Bars represent the difference in the mean time to finish (min) between present and absent trials of each habitat treatment. Positive values indicate the salmon sped up, while negative values indicate the salmon slowed down, on average when the predator was in the enclosure. *p* Values relate to the contrast between present and absent trials within habitat categories (Table 2)

Our enclosure design successfully measured salmon downstream travel speed. Most (131/144) salmon reached the PIT antenna at the end of the enclosure in the allotted 60 min, on average (± SD) in 8.5 ± 9.4 min with a distribution heavily skewed toward short times (Fig. 3). Salmon changed travel speed in response to predator presence and patterns were context dependent. Shade was the only covariate that significantly affected salmon’s reaction to predation risk (*p* = 0.002; Table 1), and shade modified both the magnitude and direction of their antipredator response (Table 2; Fig. 4). As for magnitude, salmon reacted more strongly to the predator in shade (Linear contrast [predator vs. no predator]: predator present *n* = 20, predator absent *n* = 23, *Z*-ratio = 2.72, *p* = 0.007) compared to sun (predator present *n* = 48, predator absent *n* = 53, *Z*-ratio = − 1.49, *p* = 0.14). As for direction, salmon slowed down in shade (statistically significant) and sped up in sun (non-statistically significant trend) (Fig. 4).

**Table 1:** Results of Type III ANOVA on mixed-effects Cox regression

Covariate	*df*	*χ*^2^	*P*
Predator	1	7.58	0.006*
Shade	1	15.16	< 0.001*
Structure	1	4.86	0.03*
Origin	1	2.42	0.12
Predator × shade	1	9.25	0.002*
Predator × structure	1	2.04	0.15
Predator × origin	1	0.39	0.53
Shade × structure	1	3.73	0.05^†^
Shade × origin	1	5.56	0.02*
Origin × structure	1	< 0.001	0.99

*p < 0.05

^†^*p* < 0.1

**Table 2:** Summary of pair-wise linear contrasts and effect sizes

Treatment	Predator contrast		Linear contrast		Effect size	
	Predator present (*n*)	Predator absent (*n*)	*Z*-ratio	*p*	Hedge’s *g*	Magnitude°
Shade—no structure	10	5	2.80	0.005*	1.07	Large
Shade—structure	13	15	1.97	0.048*	0.42	Small
Sun—no structure	27	31	− 0.20	0.85	− 0.09	Negligible
Sun—structure	21	22	− 1.94	0.053^†^	− 0.21	Small
Shade—hatchery	10	9	2.65	0.008*	1.01	Large
Shade—wild	13	11	2.24	0.03*	0.20	Negligible
Sun—hatchery	25	27	− 0.75	0.45	− 0.08	Negligible
Sun—wild	23	26	− 1.55	0.12	− 0.20	Negligible

Describes how, for various habitat and salmon origin treatments, predator presence changes salmon speed. *n* Represents the number of trials. All predator present and absent trials of first four rows equal 144 total trials, and likewise for the last four rows

°Magnitude of effects interpreted following the thresholds |*g*|< 0.2 “negligible”, |*g*|< 0.5 “small”, |*g*|< 0.8 “medium”, |*g*|> 0.8 “large” (Cohen 1992)

**Fig. 5:**
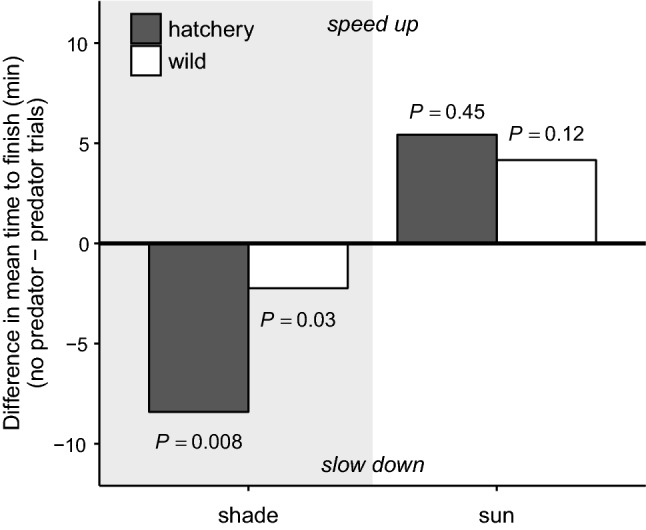
Bars represent the difference in the mean time to finish (min) between present and absent trials in the shade and sun. Dark bars represent hatchery salmon while white bars represent wild salmon mean behavior. Positive values indicate the salmon sped up, while negative values indicate the salmon slowed down, on average when the predator was in the enclosure. *p* Values relate to the contrast between present and absent trials within shade and salmon origin categories (Table 2)

Because shade was the only significant variable that interacted with predator presence, we subsequently examined the effects of structure and salmon origin within shade and sun categories (Table 1). On average, structure dampened the reaction to the predator by 7.2 min in shade (a difference between a large and small effect size) and increased the reaction by 3.6 min in sun (a difference between negligible and small effect size; Fig. 4b; Table 2). Also contrary to our expectations, hatchery and wild salmon reacted similarly to the largemouth bass—both groups slowed down in shade and sped up in sun (Table 2; Fig. 5). However, hatchery salmon slowed travel speed by 6.2 min more than wild salmon in shade with a larger effect size (Table 2).

## Discussion

In this study, the presence of shade influenced salmon antipredator behavior in the migration corridor, more than habitat structure and salmon origin. Shade affected salmon antipredator behavior in two ways. First, salmon reacted more strongly to the predator in shade than in direct sun. Second, shade affected salmon’s escape tactic—when faced with the predator, salmon slowed down in shade and sped up in sun. These patterns were consistent across hatchery and wild salmon. Habitat structure did not significantly modify prey’s perception of predation risk. These results suggest that environmental risk cues can affect both the magnitude and escape tactic of antipredator behavior, and in the migration corridor where prey have a propensity to travel directionally, these metrics can be observed via a change in travel speed with and without a predator present.

To examine context-dependent antipredator behavior in the migration corridor, we used large field mesocosms. Experiments are important to evaluate specific hypotheses, but difficult to perform on actively migrating animals (Marra et al. 2015). Experiments conducted on migratory prey in laboratory settings preclude a key component of migrant biology—the propensity to travel towards their destination (Able 1993; Pomeroy et al. 2006). We created large field enclosures that allowed salmon to travel downstream, while still allowing us to manipulate habitat and predator treatments to test our hypotheses. Despite their value in hypothesis-testing, experiments never fully capture the complete set of natural behaviors and processes. In our study, salmon could not use all possible antipredator behaviors, such as schooling, movements perpendicular to flow, or hiding in substrate (Steel et al. 2013; Furey et al. 2016). However, salmon were able to moderate activity levels, hide, and inspect the predator, which are all common antipredator responses in fish (Kelley and Magurran 2003). We did not anecdotally observe largemouth bass to actively pursue salmon, suggesting that our predators may not have exhibited threatening behavioral cues (Bateman and Fleming 2011). However, largemouth bass are an important predator and plastic models have elicited behavioral responses in salmon (Sabal et al. 2020). Many laboratory assays use single cues or model predators, and by using a live predator, we ensured that realistic visual and olfactory predator cues were present (Reinhardt and Healey 1997; Lönnstedt and McCormick 2011). The experimental conditions (e.g., enclosure, handling) could have increased the perceived vulnerability of salmon over natural conditions affecting overall movement behavior, but were consistent across predator treatments, which is how we compared behavior to address our hypotheses.

In the migration corridor, juvenile salmon reacted the most strongly to the largemouth bass when in shade. The larger behavioral reaction suggests that salmon perceived shade to be a riskier environment than sun and could be driven by reduced visibility benefitting the predator in detection or capture (Cerri 1983; McMahon and Holanov 1995). Alternatively, salmon could have been compensating for low visibility reducing information by engaging in more antipredator behavior (Metcalfe 1984; Leahy et al. 2011). In our experiment, shade was created by trees on the leveed bank, which is similar to overhead or canopy shade rather than shade of in-water structure. Similarly, salmon avoid passing under anthropogenic structures because low light decreases prey vision and acquired information (Kemp et al. 2005; Leahy et al. 2011; Ono and Simenstad 2014). In our experiment, salmon had the largest reaction to the predator in shade with no structure (large effect size) and a smaller reaction in shade with structure (small effect size). This suggests a possible interaction between shade, structure, and predator presence, which deserves further study. In other circumstances when shade and structure are highly correlated, these conditions may indicate low risk. In upstream habitats, rivers are small and bordered by riparian vegetation, so shade is often correlated with in-stream structure and typically indicates low risk (McMahon and Hartman 1989; Penaluna et al. 2015). Migrating salmon also preferentially hold in river reaches with complex habitat including shade with in-water structure (e.g. woody debris, submerged aquatic vegetation) (Zajanc et al. 2013).

Not only did shade affect the magnitude of the reaction to predation risk, but shade also affected what escape tactic juvenile salmon used. Cautious or cryptic antipredator behavior in migrating animals slows travel speed (Chung et al. 2009; Melnychuk and Welch 2018), while fleeing past predators causes prey to speed up (Peterson 1976; Proffitt et al. 2009). In our study, juvenile salmon slowed down in shade and sped up in direct sun in response to the predator. In the shade salmon may have used cautious or cryptic antipredator behavior, such as predator inspection, reduced movement, and hiding (Kelley and Magurran 2003). These behaviors may be advantageous in shade because low light decreases the detection distance between predator and prey, thereby allowing salmon to be cryptic before they are first detected by the predator (Howick et al. 1983; Mazur and Beauchamp 2003).

In contrast, salmon may have been immediately detected by the largemouth bass in direct sunlight, thereby reducing the success of slowing down to be cryptic. When in sun salmon exhibited a trend to speed up in response to the predator (although not statistically significant). The weaker reaction and larger behavioral variation to predation risk in sun suggests that salmon may have perceived the sun-lit environment as relatively low risk. Sunlight allows prey to gather information and characteristics such as predator gaze, orientation, and approach speed, which indicate the likelihood and severity of a predator attack (Cooper 2008; Bateman and Fleming 2011). The largemouth bass in our experiment actively swam up and down the enclosure, but we never anecdotally observed them actively pursue a salmon. Perhaps salmon in direct sunlight could determine that the predator was not a major threat in that moment, and therefore, salmon did not significantly alter their behavior. Our study precludes examination of salmon responses to partial shade because we considered percent shade as a binary category (shade, sun) to ensure sufficient sample sizes across treatments. Therefore, we may have missed biologically-relevant nuances between predator and prey along the partial shade gradient and related to the location of predator and prey relative to shade and sun (McCartt et al. 1997). In our study, salmon shifted escape tactics between shade and sun related to predator presence.

Shade affected the magnitude and direction of the reaction to a predator, and both patterns were consistent across hatchery and wild salmon. Hatchery salmon often fail to appropriately react to predators (Leduc et al. 2007; Jackson et al. 2011; Solberg et al. 2020). Therefore, we predicted that hatchery salmon would react less than wild salmon to predator presence due to their lack of prior predator exposure. Contrary to our expectations, hatchery salmon reacted similarly to wild salmon and reacted more strongly to the predator in shade. There is some evidence for innate antipredator responses to predator odor cues in juvenile salmon and responses to shade in hatchery bluegills (*Lepomis macrochirus*) (McCartt et al. 1997; Hawkins et al. 2007, 2008). Hatchery salmon may have innately responded to risk cues in our experiment. Wild salmon associate predator encounters with many cues including predator characteristics and habitat associations. In our experiment, wild salmon may have been better at accurately assessing local predation risk and could tell the predator was not actively foraging. This information could have allowed salmon to take advantage of the tradeoff between predation risk and other activities by avoiding behavioral changes more than necessary. The consistent patterns in antipredator behavior and shade across hatchery and wild salmon emphasize the importance of shade to modifying predation risk.

We were surprised that shade was more important and consistent in affecting salmon antipredator responses over habitat structure. Structure strongly and regularly decreases predation risk to diverse prey (Crowder and Cooper 1982; Stellatelli et al. 2015; Bonnot et al. 2016). However, resident prey are familiar with their surrounding environments, while migrating prey travel though relatively unfamiliar migration corridors (especially first time migrants). Perhaps, in the migration corridor, unfamiliar structure is less certain whether it indicates low or high risk compared to shade. In familiar environments, the pattern is often opposite—structural cues of low risk elicit stronger antipredator responses over visibility-related risk cues (Diehl 1988; Ajemian et al. 2015). However, structure indicating low risk depends strongly on specific knowledge of refuge location and safety. For example, eastern chipmunks (*Tamias striatus*) quickly retreat to burrows in familiar environments, but in unfamiliar environments, they do not know the location of burrows and instead take longer to run up trees (Clarke et al. 1993). Shade may consistently affect visibility, and, therefore, be less affected by local knowledge. For migrating prey, unfamiliar environments could change the relative importance among different risk cues and this idea deserves further study (Németh and Moore 2007; Bazazi et al. 2011; Sabal et al. 2021).

Our study suggests that shade is important in modifying the magnitude and tactics of antipredator behavior for juvenile salmon in the migration corridor. Furthermore, because antipredator behaviors in the migration corridor relate to travel speed, there is potential for these fine-scale decisions to scale up and affect larger patterns in migratory behavior. For example, decisions to temporarily hide or move nocturnally can delay arrival to the migration destination (Melnychuk and Welch 2018). Conversely, decisions to speed up to reduce predator encounters can shorten migration duration or cause early arrival (Hebblewhite and Merrill 2007). As optimal arrival time is often narrow for migratory prey, delays or advances to arrival timing may have important population consequences (Gienapp and Bregnballe 2012). Therefore, fine-scale decisions in the migration corridor, how they relate to movement, and how they may scale up to migratory patterns are valuable future research directions. Overall, this study expands our knowledge about antipredator behavior in the migration corridor by showing how environmental risk cues can shape the magnitude and tactics of how migrants react to predation risk and how these responses relate to movement.
